# Assessing the Impact of a Missed Mass Drug Administration in Haiti

**DOI:** 10.1371/journal.pntd.0000443

**Published:** 2009-08-25

**Authors:** Kimberly Y. Won, Madsen Beau de Rochars, Dominique Kyelem, Thomas G. Streit, Patrick J. Lammie

**Affiliations:** 1 Division of Parasitic Diseases, Centers for Disease Control and Prevention, Atlanta, Georgia, United States of America; 2 Hôpital Ste Croix, Leogane, Haiti; 3 Task Force for Child Survival and Development, Emory University, Decatur, Georgia, United States of America; 4 Department of Biological Sciences, University of Notre Dame, Notre Dame, Indiana, United States of America; Michigan State University, United States of America

Lymphatic filariasis (LF) is a disfiguring and debilitating parasitic disease that is endemic in 81 countries, placing a staggering 1.3 billion people at risk for filarial infection [1]. In 1997, the World Health Assembly resolved to eliminate LF as a public health problem, and in 2000, the Global Programme to Eliminate Lymphatic Filariasis (GPELF) was officially launched. Coupled with the development of essential diagnostic tools, the primary strategy devised to achieve LF elimination was to implement annual mass drug administration (MDA) using combinations of albendazole plus either diethylcarbamazine or ivermectin for at-risk populations [2]. These single-dose treatment regimens were chosen for their ability to significantly reduce microfilaremia for periods of up to one year, limiting the transmission potential. Through generous donations of drugs from GlaxoSmithKline and Merck & Co., the global program began its first treatments in 2000. Since then, 48 of the 81 endemic countries have implemented MDA and almost 2 billion treatments have been provided [1]. These treatments have led to dramatic reductions in microfilaremia and have provided significant collateral benefit by reducing soil-transmitted helminthiasis [3],[4]. Furthermore, more than 6 million cases of hydrocele and 4 million cases of lymphedema have been prevented in the last eight years, translating into more than 32 million disability-adjusted life years averted [3]. Through the efforts of a national program, China became the first country to declare the elimination of LF as a public health problem, and in March 2008, the Republic of Korea also made a similar announcement [1].

Although significant progress has been made since the inception of GPELF, there are still many challenges that stand in the way of success. At the onset of the global program, the recommended strategy was to administer annual MDA for four to six years. At that time, this recommendation was thought to be sufficient for interrupting transmission. As the program has progressed, it has become increasingly evident that although transmission appears to have been interrupted in some areas after only five rounds of MDA, this has not been the case in other places [5],[6]. Differences such as vector–parasite complexes, initial infection prevalence, and urban versus rural settings make it difficult to design each elimination program in an identical manner, and it is becoming clear that MDA works better and more efficiently in some areas than in others. For example, especially important for MDA strategies to be effective is the requirement for adequate coverage and compliance among eligible populations. Many programs have struggled to achieve the recommended 80% coverage in part because of differences in drug-delivery strategies. However, the single greatest challenge to programs may be the difficulty or inability to reach a national scale. Many countries are forced to make difficult decisions about public health allocations with limited financial resources available and are, of necessity, heavily dependent on external support to maintain their national LF programs. During periods of difficult funding, a number of programs have been forced to cut back or skip MDAs altogether. However, there has been very little effort to look at the impact of missed MDA cycles on program outcomes.

Haiti represents approximately 78% [7] of the LF burden in the Americas, and the LF program in Haiti is one that faces many challenges. The National Program to Eliminate LF in Haiti began in 2001. Based on initial mapping results, communes were stratified into high, moderate, and low priority for treatment. Because of limited resources, the decision was made to focus on MDA in the highest-priority settings, which represented areas with the greatest public health need (determined by antigen prevalence). The program scaled up rapidly, and by 2005, 18 of 20 high-priority communes (based on initial mapping; the commune of Port au Prince was subsequently subdivided) had been treated with a combination of diethylcarbamazine and albendazole at least once. Of communes with the greatest treatment need, only Port au Prince and Gonaives, which have challenging political and urban environments, were not included.

Although the national program didn't begin until 2001, a pilot mass treatment program began in Leogane the previous year. The commune of Leogane comprises both urban and rural zones and is located approximately 30 km west of Port au Prince, with an estimated population of 150,000. Before intervention, antigenemia in this area was approximately 50% [6],[8]. Nearly one year was spent on community mobilization and training for community health workers, and distribution posts were selected to provide convenient access for the communities being treated. Annual treatment with a combination of diethylcarbamazine and albendazole was implemented, and four sentinel sites were selected according to WHO guidelines to monitor the progress of the program [9]. The reported coverage for the first five rounds of MDA ranged from 50% to 104%; the surveyed coverage was 71% and 79% in 2000 and 2002, respectively [6] (unpublished data).

After five rounds of MDA (2000–2005), antigen prevalence had decreased significantly, from nearly 50% to 15%. There was also a significant reduction in microfilaremia from 15% to <1% [6]. Although great progress had been made after five rounds of MDA, it was evident that additional rounds of treatment were necessary. MDA was carried out in October 2005; however, 2006 was a challenging year for the LF program in Haiti. There was great civil strife in the country, and this instability led to decreased donor confidence. In the midst of the turmoil, it was difficult to convince donors that the LF program would be able to operate at the same level as in the years past. As a result of this decreased confidence, funding was interrupted and there was no MDA either in Leogane or in the other communes where MDA had been carried out. With the resumption of program activity in 2007, surveys were conducted in two of the sentinel sites in Leogane (Leogane town and Masson-Mathieu), nearly two years after the most recent MDA, giving insight into the impact of the missed MDA.

**Figure 1:**
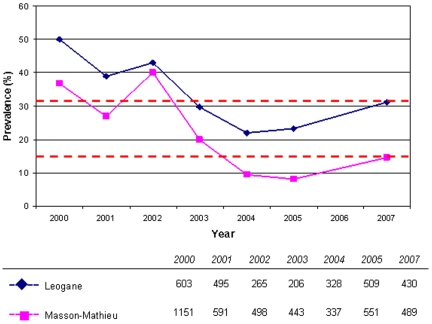
Immunochromatographic test prevalence in Leogane, Haiti (2000–2007). Antigen prevalence increased from 23.2% to 31.2% in Leogane town and from 8.2% to 14.5% in Masson-Mathieu in 2007. There was no MDA in 2006. The dashed lines indicate antigen prevalence in 2007. The total number of persons surveyed each year is indicated in the legend.

In 2007, antigen prevalence in Leogane town and Masson-Mathieu was 31.2% and 14.5%, respectively. Both findings represented significant increases (*p*<0.001) in prevalence from 2005, consistent with ongoing transmission in the area (Figure 1). The results of confirmatory antigen and antibody tests done on samples from immunochromatographic test–positive individuals supported the conclusion that the increase was real and not an artifact of changes in sensitivity of the immunochromatographic test. Microfilaremia also increased during this period in both sentinel sites, from 0.6% to 6.4% in Leogane town and 0.5% to 2.1% in Masson-Mathieu. However, differences in blood volume used for the slides in 2007 (60 µl versus 20 µl in 2005) make it difficult to draw direct comparisons between the years. Although the evidence strongly suggests that infection levels have recrudesced as a consequence of the missed MDA, it is important to recognize that systematic noncompliance may also have played a role. This problem has been reported previously in Leogane [10],[11], and the reservoir of infection represented by noncompliant persons may have been responsible for the apparent plateau in antigen prevalence observed in 2004 and 2005. Nonetheless, the missed MDA allowed antigen prevalence in 2007 to revert to nearly the levels found in 2003, apparently losing at least two years' worth of progress.

This limited example from Haiti begins to give insight into the consequences of missed MDAs and has broader implications for the global program. The potential for recrudescence of antigenemia is an obvious setback to national programs, and the Haitian experience has shown that there can be a significant loss of investment if programs are not sustained. In highly endemic settings such as Haiti, a funding gap can inadvertently introduce additional obstacles in an already challenging programmatic situation. There are also less obvious issues that must be considered. Inconsistent drug delivery could lead to confusion and fatigue within the communities as the necessary rounds of MDA are prolonged over additional years. These inconsistencies could result in a loss of credibility for the LF programs and ultimately lead to a decline in political commitment and support, a critical determinant of the success of programs [5]. Irregular drug delivery extends the time necessary to reduce infection levels below the necessary threshold for eliminating transmission and disease. This could allow for the selection of parasites that develop resistance to one or more of the drugs being used. Although there has been little evidence that lymphatic filarial parasites have developed drug resistance, the observation that MDA exerts selective pressure on parasite populations argues that programs should be consistent in applying drug pressure [12],[13].

With so many challenges facing LF programs, there must be an effort to stabilize annual rounds of MDA as much as possible. Achieving this stability requires sustained political and social commitment, careful planning to ensure adequate drug supply, and consistent funding. In stable political and funding climates, programs can optimize opportunities to achieve success. Perhaps the setback observed in Haiti was a result of a strategic error in the initial decision to focus on high-prevalence communes that were scattered geographically. Treatment in these areas may have led to a decrease in antigen prevalence, but the potential contribution of adjacent areas to ongoing transmission was not considered. In retrospect, it is difficult to know whether treating an entire geographical region would have been a better use of the limited resources. However, this does not obviate the need for continued, sustained funding in any case, possibly for a longer period than originally anticipated. Finances remain the greatest barrier to the expansion of LF programs, and limited resources are the reality of the day. However, strengthening partnerships can potentially maximize the use of scarce resources and promote financial stability. With extensive geographical overlap of neglected tropical diseases, there is great opportunity to link control programs and achieve program synergy [14]. Single-disease programs should capitalize on the burgeoning interest surrounding the integration of neglected tropical disease programs to create a framework for program stability and success. Despite the setback in Haiti, there should be a collective optimism that the effort to eliminate LF can be successful, through new partnerships and programmatic linkages.
